# Optimizing Dementia Care Interactions With the QUALity of Interactions Inventory (QUALII)

**DOI:** 10.1093/geroni/igaf122.1335

**Published:** 2025-12-31

**Authors:** Anju Paudel, Elizabeth Galik, Beth Howd, Marie Boltz

**Affiliations:** The Pennsylvania State University, University Park, Pennsylvania, United States; University of Maryland School of Nursing, Baltimore, Maryland, United States; The Pennsylvania State University, University Park, Pennsylvania, United States; The Pennsylvania State University, University Park, Pennsylvania, United States

## Abstract

To optimize daily care interactions and enhance quality of care and quality of life of residents with dementia, a comprehensive measure is needed that can guide the desired practice of positive care interactions and evaluate the impact of practice. Such a comprehensive measure of staff-resident interactions in dementia care in assisted living (AL) settings is lacking. Thus, we developed the QUALity of Interactions Inventory (QUALII)— a five domain 21-item comprehensive tool with items representing approaches to positive care interaction in dementia care in AL. This study aims to describe the development and refinement of QUALII. QUALII was developed based on communication theory and the review of the literature. It was revised with input from a panel of N = 10 clinical and academic experts in dementia care and communication utilizing the Delphi survey approach. Following three rounds of Delphi survey, the five domain 21-item QUALII was reduced to a five domain 19-item tool. The revised QUALII demonstrated good content validity based on Content Validity Index (CVI) of individual items (Item relevance I-CVI range= 0.90 to 1.00; Item clarity I-CVI range= 0.80 to 1.00) and the overall scale (S-CVI Average= 0.93). The tool’s theoretical foundation, evidence base, and demonstration of content validity suggests its significant potential to guide and evaluate positive care interactions in dementia care and warrants further psychometric testing.

